# Mild Traumatic Brain Injury Results in Significant and Lasting Cortical Demyelination

**DOI:** 10.3389/fneur.2022.854396

**Published:** 2022-06-23

**Authors:** Sean O. Mahoney, Nahian F. Chowdhury, Van Ngo, Phoebe Imms, Andrei Irimia

**Affiliations:** ^1^Ethel Percy Andrus Gerontology Center, Leonard Davis School of Gerontology, University of Southern California, Los Angeles, CA, United States; ^2^Corwin D. Denney Research Center, Department of Biomedical Engineering, Andrew and Edna Viterbi School of Engineering, University of Southern California, Los Angeles, CA, United States

**Keywords:** neuroimaging, cortex, myelin, concussion, neurodegeneration, Alzheimer's disease, mild traumatic brain injury

## Abstract

Despite contributing to neurocognitive deficits, intracortical demyelination after traumatic brain injury (TBI) is understudied. This study uses magnetic resonance imaging (MRI) to map intracortical myelin and its change in healthy controls and after mild TBI (mTBI). Acute mTBI involves reductions in relative myelin content primarily in lateral occipital regions. Demyelination mapped ~6 months post-injury is significantly more severe than that observed in typical aging (*p* < 0.05), with temporal, cingulate, and insular regions losing more myelin (30%, 20%, and 16%, respectively) than most other areas, although occipital regions experience 22% less demyelination. Thus, occipital regions may be more susceptible to primary injury, whereas temporal, cingulate and insular regions may be more susceptible to later manifestations of injury sequelae. The spatial profiles of aging- and mTBI-related chronic demyelination overlap substantially; exceptions include primary motor and somatosensory cortices, where myelin is relatively spared post-mTBI. These features resemble those of white matter demyelination and cortical thinning during Alzheimer's disease, whose risk increases after mTBI.

## Introduction

Approximately 1.7 million Americans sustain traumatic brain injuries (TBIs) every year (1), **~**75% of which are mild TBIs (mTBIs). TBI can cause cerebral demyelination, mechanical damage to axons, and impairment of remyelination mechanisms resulting in either insufficient or excessive remyelination (2). These phenomena can contribute to secondary traumatic axonal injury, oligodendrocyte dysfunction, and/or brain cell death (3). Together, such events can lead to macroscale brain structure changes associated with cognitive deficits (4), mild cognitive impairment (MCI) (5, 6), Alzheimer's disease (AD) (7) and other neurological disorders (8).

Whereas both acute and chronic post-traumatic demyelination of the cerebral cortex have been studied extensively in *animals* (9–11), hardly any *human* studies have quantified the spatial profiles and extent of these phenomena. This is due partly to the challenges of measuring human cortical myelin content *in vivo*. The gold standard for measuring human cortical myelin content is *post-mortem* histopathological examination. However, as Glasser & Van Essen (12) showed, the ratio *R* of *T*_1_- to *T*_2_-weighted magnetic resonance images (MRIs) provides an accurate measure of brain myelin content because *T*_1_-weighted MRI intensity and the reciprocal of *T*_2_-weighted MRI intensity are both proportional to myelin content. Computing *R* offers improved myelin estimates because this measure implicitly adjusts for nuisance effects that can contribute to the intensities of each distinct MRI modality (13, 14).

Studies have confirmed the reliability of the *T*_1_/*T*_2_-weighted MRI intensity ratio *R* as a trustworthy measure reflecting relative intracortical myelin content after TBI (15). Specifically, in TBI subjects, cortical maps of *R* are not only consistent with maps of myelin content inferred from histopathology, but also confirmatory of the fact that TBI is associated with relatively lower cortical myelin content in cross-section (15). To our knowledge, no detailed longitudinal study of post-traumatic cortical myelin change has been published.

Due to the potential to stimulate remyelination before axonal injury occurs, myelination-related therapies are being investigated to improve mTBI outcomes (16). Furthermore, better understanding of the effects of mTBI on brain architecture can improve surgical intervention (17, 18). For these and other potential reasons, the study of post-traumatic demyelination is clinically relevant. This study quantifies how the *T*_1_/*T*_2_ MRI intensity ratio *R* of the gray matter (GM) changes across the first ~6 months after mTBI relative to age- and sex- matched healthy controls (HCs). Whereas other studies have mapped *R* after TBI, this is the first study that leverages MRI to quantify post-traumatic cortical myelin *changes* and to map the *spatial patterns* of these changes.

## Methods

### Participants and Data Acquisition

This study was conducted with the approval of the Institutional Review Board at the University of Southern California and in compliance with both the Declaration of Helsinki and the US Code of Federal Regulations (45 C.F.R. 46). Written informed consent was provided by all participants. A total of 80 HCs scanned at two timepoints were included from the Alzheimer's Disease Neuroimaging Initiative (ADNI; http://adni.loni.usc.edu). ADNI was launched in 2003 as a public-private partnership, led by Principal Investigator Michael W. Weiner, MD. The primary goal of ADNI has been to test whether serial MRI, positron emission tomography (PET), other biological markers, and clinical and neuropsychological assessment can be combined to measure the progression of MCI and early AD. For up-to-date information, see www.adni-info.org. Demographics of the HC cohort are listed in Table 1. The APOE genotypes were ε2/ε3 (*N* = 9 participants, i.e., 11% of the HC sample), ε2/ε4 (*N* = 1, i.e., 1%), ε3/ε3 (*N* = 47, i.e., 59%), ε3/ε4 (*N* = 22, i.e., 28%), and ε4/ε4 (*N* = 1, i.e., 1%). *T*_1_-weighted MRIs were acquired using a magnetization-prepared rapid acquisition gradient echo sequence (repetition time (*T*_*R*_) = 2,400 ms; inversion time (*T*_*I*_) = 1,000 ms; percentage sampling = 100; matrix size = 192 × 192; slice thickness = 1.2 mm). *T*_2_-weighted MRIs were acquired using a spin-echo sequence (*T*_*R*_ = 3,000 ms; *T*_*E*_ = 100 ms; slice thickness = 3.0 mm). A total of 70 HC subjects were scanned using a GE-manufactured scanner, while 10 were scanned using a Siemens-manufactured scanner. The myelin mapping procedure used here is valid only for examination of the cortex, whereas diffusion weighted imaging (DWI) is less accurate in GM than in white matter (WM) due to the relatively higher isotropy of water diffusion within GM (19). For this reason, DWI data were not included here. All participant data were deidentified and delinked.

**Table 1:** Age demographics of HC and mTBI cohorts.

	**Units**	**HC**	**mTBI**
*N*		80	97
males	%	53	63
μ	Yr	75.4	43.1
σ	Yr	54.7	187.9
min	Yr	60.0	19.0
max	Yr	90.0	79.0
mean ISI	Yr	3.3	0.5

*HC, healthy controls; mTBI, mild traumatic brain injury; ISI, interscan interval*.

The mTBI group included 97 participants whose scans had been obtained both acutely (7.4 ± 3.2 days post-injury) and chronically (5.75 ± 0.3 months post-injury). Demographics of the mTBI cohort are listed in Table 1. For inclusion, mTBI volunteers had (a) one mTBI caused by a fall, (b) no clinical findings on acute *T*_1_/*T*_2_-weighted MRIs, (c) no clinical findings except cerebral microbleeds on susceptibility weighted imaging (a type of MRI on which hemorrhages and other iron-rich brain deposits are hypointense), (d) an acute Glasgow coma score greater than 12 upon initial medical evaluation, (e) loss of consciousness of fewer than 30 minutes, (f) post-traumatic amnesia of fewer than 24 hours, and (g) no clinical history of pre-traumatic neurological disease or disorders, including dementia and MCI, psychiatric disorder and drug/alcohol abuse. *T*_1_-weighted MRIs were acquired using a magnetization-prepared rapid acquisition gradient echo sequence (repetition time (*T*_*R*_) = 1,950 ms; echo time (*T*_*E*_) = 3 ms; inversion time (*T*_*I*_) = 900 ms; percentage sampling = 100; matrix size = 256 × 256; voxel size = 1 mm × 1 mm × 1 mm). *T*_2_-weighted images were acquired using a turbo spin-echo sequence (*T*_*R*_ = 3,200 ms; *T*_*E*_ = 222 ms; sampling percentage = 100; matrix size = 128 × 128; voxel size = 2 mm × 2 mm × 2 mm). All mTBI subjects were scanned using a Siemens-manufactured scanner. All participant data were deidentified and delinked. *T*_1_- and *T*_2_-weighted MRIs were acquired from each mTBI participant during two sessions held at least ~5 months apart. mTBI subjects did not belong to the ADNI cohort.

### MRI Preprocessing

Although calculating the ratio *R* of *T*_1_-to-*T*_2_ image intensities facilitates the attenuation of receive field ($B_{1}^{-}$) inhomogeneities that are inherent to *T*_1_ and *T*_2_ MRIs (12), bias field correction remains useful for alleviating remaining inhomogeneities and for improving the uniformity of MRI sensitivity to myelin content across image sequences (14, 20). For these reasons, SPM12 software was used with default parameters to correct intensity inhomogeneity artifacts due to transmit fields, as detailed elsewhere (21). *T*_1_ and *T*_2_ volumes acquired during follow-up visits were registered rigidly to the *T*_1_ and *T*_2_ volumes acquired at baseline. The BRAINSFit algorithm (22) available in 3D Slicer (http://slicer.org) was used to apply successive co-registrations that improved the accuracy of the overall alignment procedure. These included (A) a rigid registration (6 degrees of freedom, df), (B) a rigid registration with scaling (7 df), (C) a rigid registration followed by scaling and shearing (10 df), (D) a fully affine registration (12 df), and (E) a *B*-spline registration with more than 27 df. To reduce registration errors, the transformation matrices associated with each registration were computed sequentially, multiplied across, and then applied to the MRI volumes as a single transformation performed in one step. By default, the percentage of image voxels sampled by BRAINSFit to determine the number and types of co-registrations is 0.2%. Here, this percentage was increased to 10% to improve precision.

### MRI Segmentation

*T*_1_- and *T*_2_-weighted volumes were resliced to 1 mm^3^ isotropic voxel resolution and segmented using FreeSurfer (FS) 6.0 software with default parameters, as detailed elsewhere (23, 24). FS operations include (A) removal of non-brain tissue, (B) GM segmentation, (C) tessellation of the WM/GM boundary to generate triangular meshes of the meshes associated with the pial surface and with the GM/WM interface surface, and (D) surface topology correction. The quality of *T*_1_-weighted volume segmentations were enhanced by including *T*_2_-weighted volumes in the segmentation to improve pial surface reconstruction, the removal of tissue outside the brain, and the co-registration of volumes acquired using the two modalities. Regional cortical thickness and volumes were calculated by FreeSurfer and normalized using each subject's total intracranial volume. This normalization procedure removed the confound of head size, thereby enabling comparison across subjects.

### Estimation of Relative Myelin Content

The ratio *R* of *T*_1_-to-*T*_2_-weighted volume intensities was calculated for each voxel after the volumes acquired at each wave had been co-registered within the same coordinate space. The FS GM mask was used to identify *R* values associated with GM voxels. The meshes representing the GM/WM interface surface and the pial surface were used for calculations that assigned an *R* value to each vertex on the GM/WM surface. The procedure resembled the one used by FS to calculate cortical thickness partly because correspondences between pial mesh vertices and WM mesh vertices were identified. These correspondences allowed us to compute, for each WM mesh vertex, the shortest distance between that vertex and the pial surface. Then, the line segment specifying the distance between the two surfaces was computed, subject to topological constraints to ensure proper pairing of vertices on the WM mesh to vertices on the pial surface mesh. For each such pair of vertices, the equation of the straight line that passed through the line segment connecting the two vertices was calculated. The value of the myelin measure *R* assigned to each vertex on the GM/WM surface was computed as a weighted average of the *R* values at the voxels traversed by this line segment. The averaging accounted for the number of GM voxels traversed and for the relative proportion of the line segment that lay within each of these voxels. For inter-subject averaging purposes, maps of *R* were co-registered to the FS average atlas.

### Cognitive Measures Relative to *R*

Cognition was evaluated at approximately 2 weeks and 6 months post-injury in the mTBI cohort using the Brief Test of Adult Cognition by Telephone (BTACT) (25). The BTACT is a convenient phone-based cognitive assessment, comprising six classic measures of episodic verbal memory [both immediate (EVMI) and delayed (EVMD)], working memory span (WMS), processing speed (PS), verbal fluency (VF), and inductive reasoning (IR). Details on the administration, interpretation, and psychometric properties of these tests can be found elsewhere (26). Cognitive scores for the mTBI cohort are listed in Table 2.

**Table 2:** Cognitive scores for traumatic brain injury subjects, at ~2 weeks and 6 months post injury (baseline and follow-up, respectively).

**Baseline**	**μ**	**σ**
EVMI	3.46	2.82
EVMD	2.24	2.26
WMS	3.69	1.57
VF	12.41	6.99
IR	1.40	1.24
PS	27.20	13.95
**Follow-up**	**μ**	**σ**
EVMI	5.06	2.67
EVMD	2.83	2.45
WMS	4.52	1.61
VF	16.72	7.95
IR	2.10	1.50
PS	32.63	14.85

*EVMI, episodic verbal memory immediate; EVMD, episodic verbal memory delayed; WMS, working memory span; VF, verbal fluency; IR, inductive reasoning; and PS, processing speed; ant. TTG, anterior transverse temporal gyrus; ACC, anterior part of the cingulate gyrus and sulcus*.

Prior to analysis, mTBI cognitive scores *x* were converted to *z*-scores as *z* = (*x* − μ)/σ relative to the mean μ and standard deviation σ of *x* in a reference cohort comprising 4,513 healthy adults (2,027 males) aged 28–84 years (μ ± σ = 55.80 ± 12.31 years) who had participated in the Midlife in the United States (MIDUS) longitudinal study. Because BTACT cognitive scores were not normally distributed, Spearman's rank correlation was used to examine associations between *R* and cognition. We calculated correlations between (A) each measure of relative myelin content (i.e., *R* at each timepoint and the percentage change in *R* across timepoints) and (B) each cognitive variable assessed at every timepoint (EVMI, EVMD, WMS, VF, IR, and PS). Because BTACT scores are intercorrelated (27), a false discovery rate correction was implemented using the procedure of Benjamini & Yekutieli (28) for variables with dependencies.

### Statistical Model

To compare *R* across groups (mTBI participants vs. HCs) and time points (acute baseline vs. chronic follow-up), a generalized linear mixed model with repeated measures was implemented using the fitlme function in MATLAB (MathWorks, Inc., Natick, MA). This model is of the form ***y*** = ***Xβ*** + ***Zu*** + **ε**, where ***y*** is a matrix containing the observations of the dependent (response) variable *R*, modeled as a continuous random variable with repeated measures. ***X*** is the design matrix of fixed effects and **β** contains the linear regression coefficients for the fixed effects. The three factors in the fixed model are sex (nominal variable), scanner manufacturer (discrete variable; 0 = Siemens; 1 = GE), and diagnosis (ordinal variable; 0 = absence of mTBI, i.e., HC; 1 = presence of mTBI). Age (continuous random variable with Gaussian distribution) and its higher powers up to fourth order are confounding variables. To identify the most appropriate fixed-effects model, the compare function in MATLAB was used to implement stepwise forward model selection. The compare function calculates the Akaike and Bayesian information criteria of two input models and uses a likelihood ratio test to determine which model best explains the underlying data without overfitting. Based on this strategy, the variables included in ***X*** were diagnosis, age, sex, scanner manufacturer, and the age × sex interaction. Because 10 HCs had been scanned on a Siemens-manufactured scanner, the column of ***X*** coding the scanner manufacturer was not colinear with that accounting for diagnosis effects.

In our model ***y*** = ***Xβ*** + ***Zu*** + **ε**, ***Z*** is the design matrix of random effect variables accounting for effects pertaining to interscan intervals (ISIs, i.e., by the time intervals *t* between baseline and follow-up scans). These random-effect variables are modeled by normally distributed, continuous random variables grouped by subject. In the HC group, the ISI models the amount of natural aging occurring between baseline and follow-up. In the mTBI group, it additionally models injury chronicity because baseline scans are acquired shortly after traumatic events. At baseline, *t* = 0 months in both groups; at follow-up, *t* varies across subjects. To account for ISI variability across subjects without regressing out the main effect of time, the deviation Δ*t*_*i*_ = *u*(*t*)−*t*_*i*_ of the ISI *t*_*i*_ of each subject *i* from the ISI grand mean *u*(*t*) was calculated first. These deviations were included in the generalized linear mixed model as random effects. The deviations from the ISI grand mean (rather than each subject's deviation from her/his group mean) were included because the two groups have different ISI means. For this reason, accounting for deviations from the *individual* group means (rather than from the *grand* mean) would have regressed out some of the interaction between the main effects of time and diagnosis. However, by accounting for deviations from the ISI *grand* mean, the confounding effect of ISI was treated identically across both groups within the generalized linear mixed model.

The vector ***u*** contains the random effects of Δ*t* described above; the covariance matrix of ***u*** is ***G***. The mathematical object **ε** is a vector of random errors with covariance ***V***. Assuming normality, ***u*** **~** *N*(***0***, ***G***), **ε ~** *N*(***0***, ***V***) and ***cov***(***u***,**ε**) =***0***. Thus, our full model includes:

(A) The within-subject response variable *R* with repeated measures that correlates with cortical myelin content across time.
(B) The between-subject predictor variables coding for factors with fixed effects (i.e., sex, scanner manufacturer, and diagnosis).
(C) One between-subject covariate (age at injury).
(D) The between-subject interactions among fixed-effect predictor variables (e.g., sex × diagnosis, age × diagnosis, etc.).
(E) The random-effect variables, grouped by subject, accounting for ISI.

The fixed-effect portion of our model includes an intercept for the grand mean, which is part of **X*****β***. The random-effect component **Zu** of the linear model organizes, *by subject*, the relationships between Δ*t*, defined above, and *R* measurements. In this approach, the random-effect relationships are modeled by the linear equations *y*_*m*_ = *b*_0*m*_+*b*_1*m*_Δ*t*_*mp*_, where *m* is the index of each grouping variable (subject), and *m* = 1, …, *n* since there are *n* subjects. The random-effect intercept *b*_0*m*_ and slope *b*_1*m*_ have prior distributions $b_{0m}\;\sim \;N\;\left(0,\;\sigma_{0}^{2}\right)$ and $b_{1m}\;\sim \;N\;\left(0,\;\sigma_{1}^{2}\right)$, respectively. The quantity Δ*t*_*mp*_ is the ISI deviation of subject *m*, at visit *p*, from the grand mean of *t*. The observation error term ε_*im*_ has the distribution $\varepsilon_{im}\;\sim \;N\;\left(0,\sigma^{2}\right)$, where *i* = 1, …, *np*. The MATLAB fitlme function models the covariance matrix of a mixed linear system as a full matrix decomposed using Cholesky parametrization. It then uses maximum likelihood estimation to fit the unknown parameters using a trust region-based quasi-Newton optimizer with a relative tolerance of 1.0 × 10^−6^ on the gradient of the objective function, an absolute tolerance of 1.0 × 10^−12^ on step size, a maximum number of 10^5^ iterations, and with an internally defined default value to start iterative optimization. To verify solution optimality, the algorithm checks the positive definiteness of the objective function's Hessian with respect to unconstrained parameters at convergence.

### Hypothesis Testing

The probability density function of the average *u*(*R*), computed across both cortical vertices and subjects, was plotted for each diagnostic group (HC, mTBI) and timepoint. The average *u*(*R*) computed only across subjects was plotted on the cortical surface both before and after averaging across each cortical region. To quantify the size of *within-subject* effects, we first calculated Δ*R* (the change in the relative myelin content *R*) as a percentage of *R* at baseline (*R*_*b*_). Defining *R*_*f*_ as the value of *R* at follow-up, it follows that Δ*R* = (*R*_*f*_ − *R*_*b*_)/*R*_*b*_. The calculation of Δ*R* was implemented separately for HCs and for mTBI participants, while partialing out the effects of sex and age at injury. In other words, the adjusted values of *R*_*b*_ and *R*_*f*_ were obtained by regressing out the effects of both sex and age at injury. Δ*R* was subsequently computed from the age- and sex-adjusted values of *R*_*b*_ and *R*_*f*_. Of note, the random effects of ISIs were *not* partialed out when computing the adjusted values of *R*_*b*_ and *R*_*f*_ because doing so would have regressed out (much of) the random effect of time, which is of interest. Instead, to account for ISIs, the confound-adjusted value of Δ*R* was converted to a percentage change in *R* observed across the mean ISI of the mTBI group. This procedure allowed us to quantify the change in *R* relative to a reference baseline group (HCs), independently of age at injury, sex, and of each subject's ISI. The probability density function of *u*(Δ*R*) computed across both cortical vertices and subjects, was plotted for each diagnostic group. The average *u*(Δ*R*) computed only across subjects was also plotted on the cortical surface.

To facilitate comparison of HCs and mTBI participants, the values of *R* and Δ*R* were computed at each vertex of the cortical mesh, averaged across the participants in each diagnostic group, and then averaged across the cortical mesh vertices within each cortical parcel (29). The null hypothesis *u*(*R*_*TBI*_) = *u*(*R*_*HC*_) of no main effect of diagnosis on *R* was tested while partialing out the effects of all other confounding variables and their interactions. A paired-sample *t* test was used to test the null hypothesis *u*(*R*_*b*_) = *u*(*R*_*f*_) of no difference in *R* between baseline and follow-up within groups. This hypothesis was tested after regressing out the statistical effects of age at injury, sex, scanner manufacturer, and ISI. Welch's two-sided *t* test for the comparison of heteroskedastic samples was used to test the null hypothesis *u*(Δ*R*_*TBI*_) = *u*(Δ*R*_*HC*_) of no difference in Δ*R* between HCs and mTBI participants independently of age at injury, sex, scanner manufacturer, and ISI. Because the distribution parameters of HCs' ISI differed from those of mTBI participants, the expected value assumed by Δ*R*_*HC*_ across the mean ISI of the mTBI participants was interpolated from the two repeated measures of *R* observed in HCs. This interpolation was based on a linear model which assumed a constant rate of change in *R* and which yielded the expected value of Δ*R*_*HC*_ across ~6 months (i.e., the mean ISI of mTBI participants). Statistical hypotheses were tested for all cortical regions, averaged across hemispheres. All hypotheses were tested at the significance level α = 0.05. To control the family-wise error rate, corrections for multiple comparisons were implemented using a standard approach (30). The approach described above was also used for the analysis of regional changes in cortical thickness and volume.

### Visualization and Comparison of Results

The difference in myelin percent loss between the two groups (mTBI and HC) is equal to *u*(Δ*R*_*TBI*_) − *u*(Δ*R*_*HC*_), a quantity that was mapped on the cortex. To further explore these relative demyelination differences between groups, we computed the vulnerability *v* of each cortical region *r* relative to the set *S* of all other cortical regions as [*u*(Δ*R*_*r*_) − *u*(Δ*R*_*S*_)]/*u*(Δ*R*_*S*_), where Δ*R*_*r*_ is Δ*R* for cortical region *r*, and Δ*R*_*S*_ is Δ*R* across all other cortical regions, i.e. across *S*. A positive value of *v* indicates that, on average, cortical region *r* experiences *v*% more demyelination than the rest of the cortex. Due to differences in scan parameters, direct comparison of *R* values between diagnostic groups may be problematic. Instead, to investigate the acute effects of mTBI on *R* within cortical parcel *j*, we computed the *z*-score *z*_*TBI*_ = [*R*_*TBI*_ − *u*(*R*_*TBI*_)]/σ(*R*_*TBI*_) for TBI participants and similarly *z*_*HC*_ for HCs. We then calculated the between-group difference in *z*-scores Δ*z* = *z*_*TBI*_ − *z*_*HC*_ to compare mTBI participants' myelination to that of HCs. Using this procedure, we identified regions whose baseline *R* deviated excessively in mTBI subjects from its expected value in HCs. Advantageously, this approach does not assume that *R* has identical distribution parameters (*u*, σ) within each group.

### Age Sensitivity Analysis

Due to the difference in the age distributions of the groups, a sensitivity analysis was implemented to evaluate the regression model's effectiveness in partialing out age effects. All HCs younger than the oldest mTBI subject (i.e., younger than 79 y) and all mTBI subjects older than the youngest HC subject (i.e., older than 60 y) were selected to create subgroups whose participants whose ages had identical ranges. The HC subgroup consisted of 63 participants (age μ = 73 y, σ = 3 y) while the mTBI subgroup consisted of 17 participants (age μ = 71 y, σ = 4 y). The analysis described in subsection on hypothesis testing was repeated using only these subgroups.

## Results

### Myelination Findings in Health and mTBI

**Figure 1:**
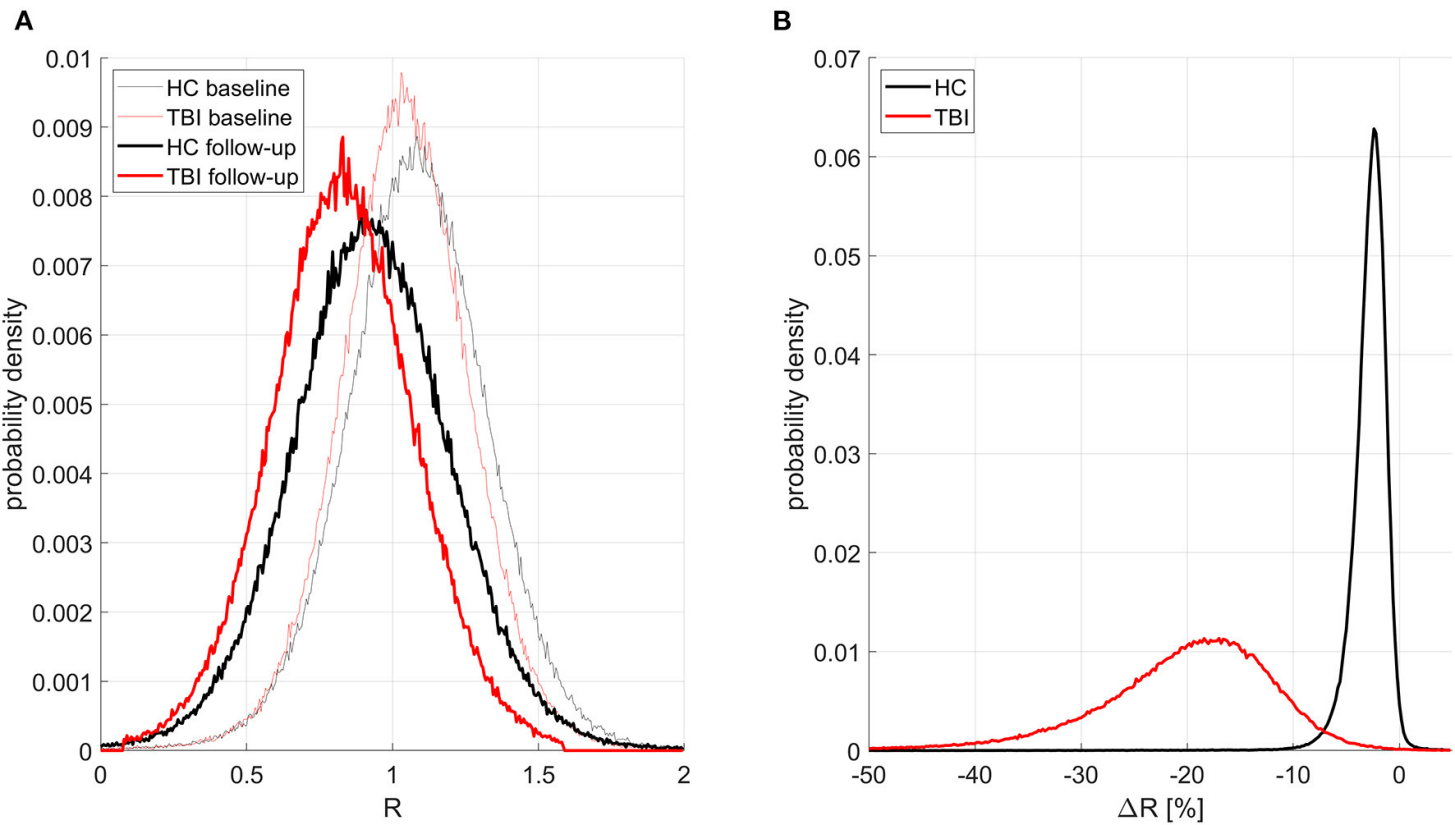
Probability distribution functions (PDFs) of the confound-adjusted myelin ratio *R* across all cortical locations for all groups and study timepoints. Traces for HCs and mTBI participants are drawn in black and red, respectively. Thinner and thicker lines correspond to timepoints 1 and 2, respectively. **(A)** PDFs for subject averages of *R* across the cortex. As expected, at both timepoints, HCs have more myelin than mTBI participants, and there is more myelin at the first timepoint in each group. **(B)** PDFs of Δ*R* (percent change in *R* across timepoints) averaged across subjects. HCs have a much smaller relative decrease in myelin with time, with a relatively small standard deviation of 0.57% in the observed percentage change. By contrast, mTBI participants experience greater myelin loss, with a substantially larger standard deviation of 2.71%.

**Figure 2:**
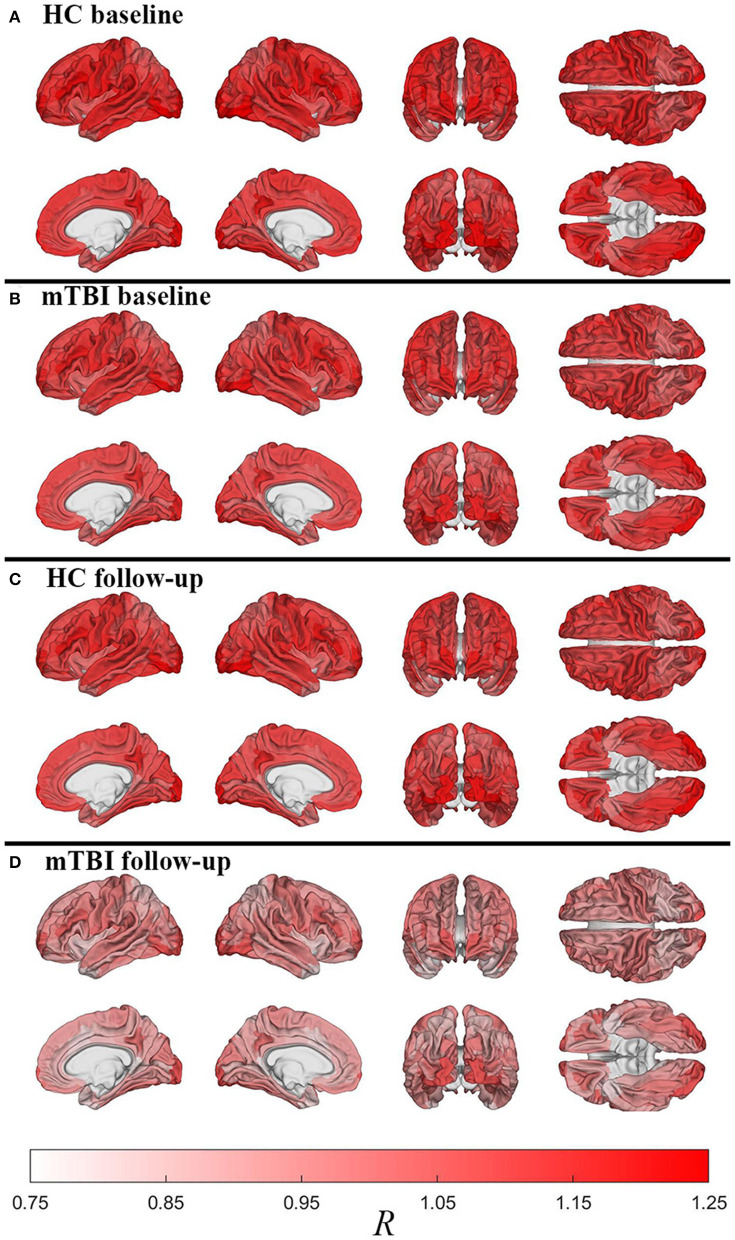
Cortical maps of the relative myelin content ratio *R* averaged over subjects within each region of FreeSurfer's cortical parcellation scheme. Shown are HCs [**(A)**: baseline; **(C)**: follow-up) and mTBI participants [**(B)**: baseline; **(D)**: follow-up). After accounting for age and sex effects, HCs have much more myelin than mTBI participants across the entire cortex, as expected based on Figure 1. Similarly, within each group, there is more myelin at the first timepoint compared to the second timepoint, even after adjusting for individual interscan intervals. However, whereas HCs experience relatively modest decreases in myelin with time, there is considerably more such loss in mTBI participants, particularly in dorsolateral frontal, lateral temporal, and especially occipital regions.

**Figure 3:**
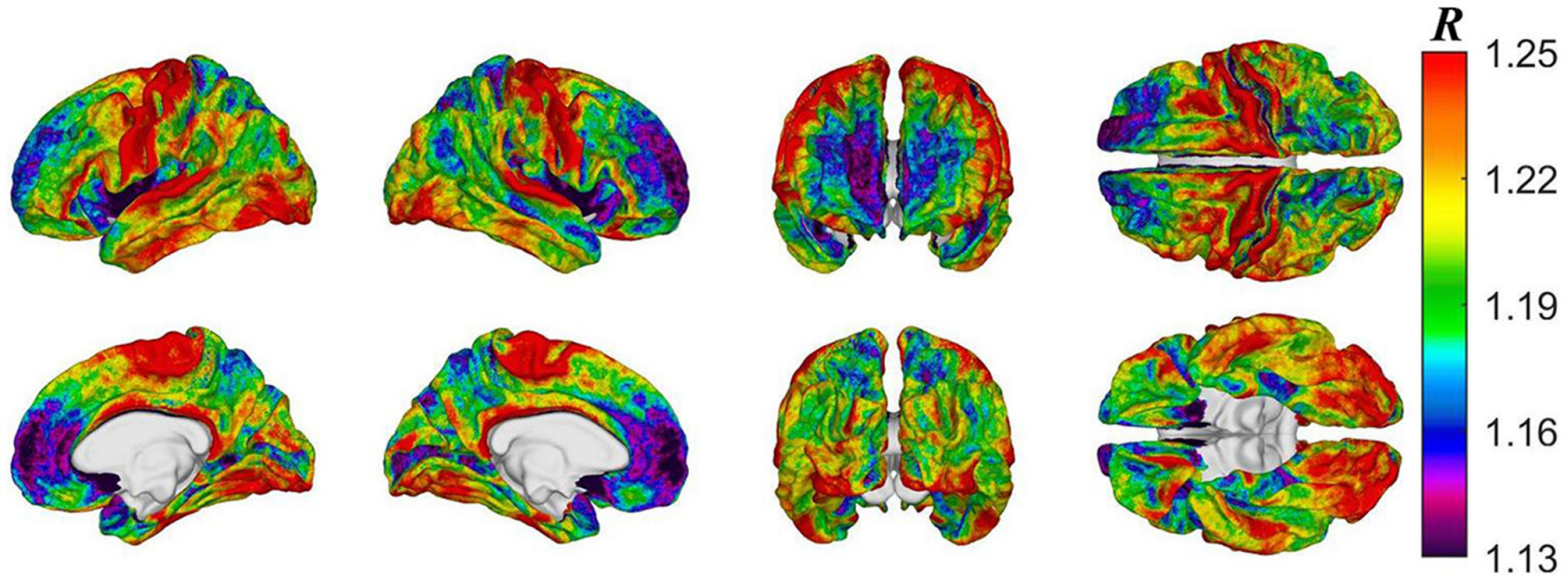
Cortical map of subject-averaged *R* in HCs at baseline. For visual comparison, the color scheme resembles that of Glasser et al. and *R* values were computed at the vertex level.

Figures 1–**3** illustrate the results obtained from the analysis described in the first paragraph of the subsection on hypothesis testing. Figure 1A displays, for all groups and timepoints, the probability density functions (PDFs), over cortical vertices, for the subject average of the confound-adjusted myelin ratio *u*(*R*). Figure 1B displays the PDFs of *u*(Δ*R*), similarly computed. In mTBI participants, on average, *u*(*R*) decreases by 20.0% across the follow-up interval; this decrease is significantly greater than that of HCs across the entire cortex (all *p* < 0.05).

Figure 2 displays cortical maps of *R* in HCs (**2A**: baseline; **2C**: follow-up) and mTBI participants (**2B**: baseline; **2D**: follow-up). Overall, cortical *R* patterns are similar across both groups and time points. The observed patterns of *R* bear similarity to those of Glasser et al. (12), who used a similar quantitative approach to map myelin content. For example, in HCs at baseline, *u*(*R*) is highest within the inferior occipital (*u*(*R*) = 1.25) and anterior transverse temporal (*u*(*R*) = 1.23) gyri, at the occipital poles (*u*(*R*) = 1.21), within precentral (*u*(*R*) = 1.16) and postcentral gyri (*u*(*R*) = 1.15), and within frontomarginal (*u*(*R*) = 1.15), subcentral (*u*(*R*) = 1.18), and transverse frontopolar gyri and sulci (*u*(*R*) = 1.19). Figure 3 displays HCs' cortical map of *u*(*R*) at baseline, using a color scheme resembling that of Glasser et al. In both groups and across both timepoints, *u*(*R*) values are relatively lower within most cingulate areas, except for the moderately myelinated dorso-posterior part of the cingulate gyrus (*u*(*R*) = 1.08). *u*(*R*) values are typically lowest in the ventro-posterior (*u*(*R*) and middle-anterior (*u*(*R*) = 1.01) parts of cingulate cortex. As expected, the insula has relatively low *u*(*R*), as do frontal and temporal lobes. The temporal pole is very lightly myelinated (*u*(*R*) = 0.96).

**Figure 4:**
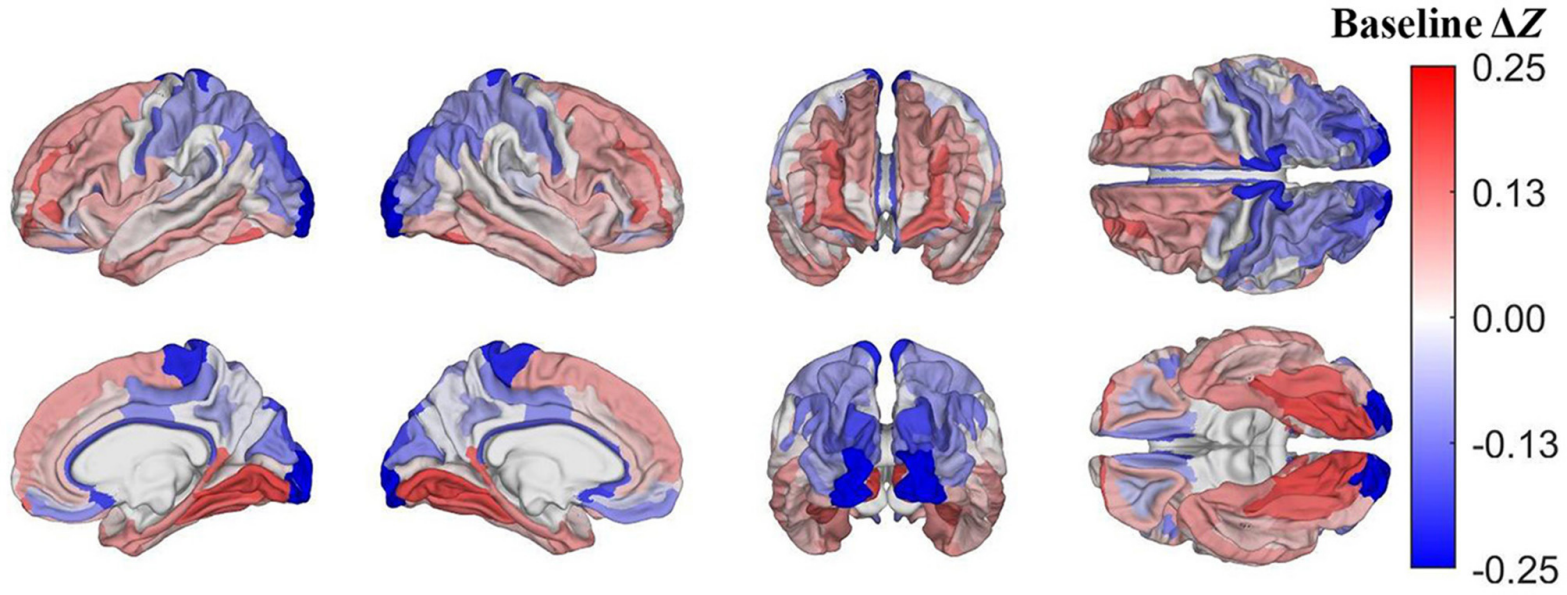
Cortical map of differences in *z*-scores of cortical regions' *R* values between mTBI and HC participants at baseline, corrected for multiple comparisons. Darker blue indicates that Δ*z* < 0, i.e., that a cortical region has a lower *z*-score in mTBI participants than in HCs. For example, at baseline, the occipital poles and paracentral lobules and sulci exhibit substantially lower *z*-scores in mTBI subjects than in HCs.

Pertaining to the analysis of acute mTBI effects described in subsection on visualization and comparison of results, on average, after accounting for age, sex, and other covariates, the largest *z*-score differences are localized to the occipital poles (Δ*z*= −0.24) and paracentral lobules and sulci (Δ*z* = −0.19, Figure 4). These regions, then, had lower *u*(*R*) values relative to *u*(*R*_*TBI*_) than expected in the HCs.

### Myelination Changes

**Figure 5:**
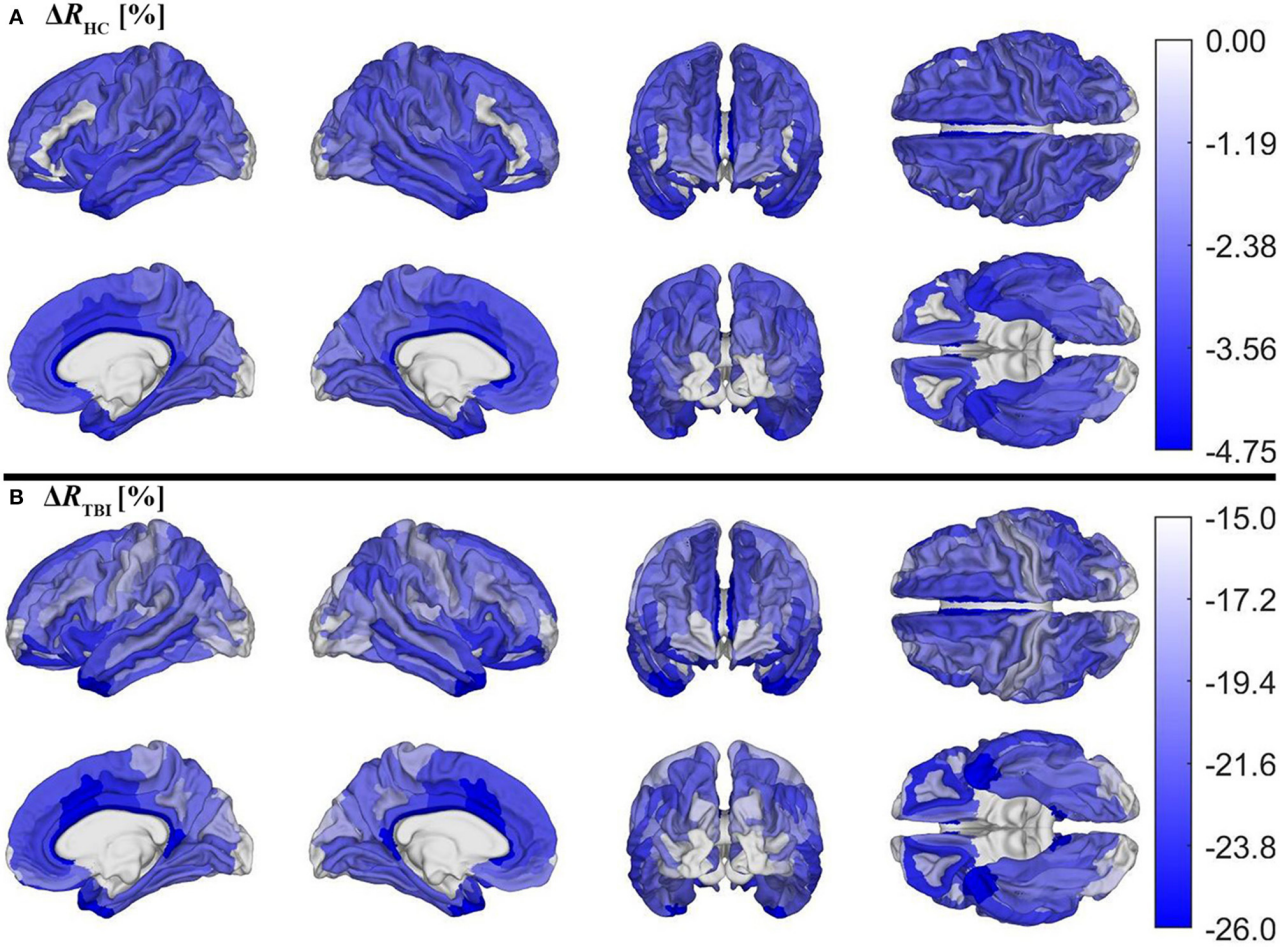
Cortical maps of the percentage change Δ*R* across timepoints for **(A)** HCs and **(B)** mTBI participants, corrected for multiple comparisons. Δ*R* was calculated as Δ*R* = (*R*_*f*_−*R*_*b*_)/*R*_*b*_, where *R*_*b*_ indicates *R* values at baseline and *R*_*f*_ indicates *R* values at follow-up. Δ*R* values were averaged over subjects and then within each region. Note that different color axes are used for each group to emphasize between-group differences across the cortex. Darker blue indicates more demyelination. Both groups lose the largest proportion of myelin in temporal, cingulate, and insular regions.

**Figure 6:**
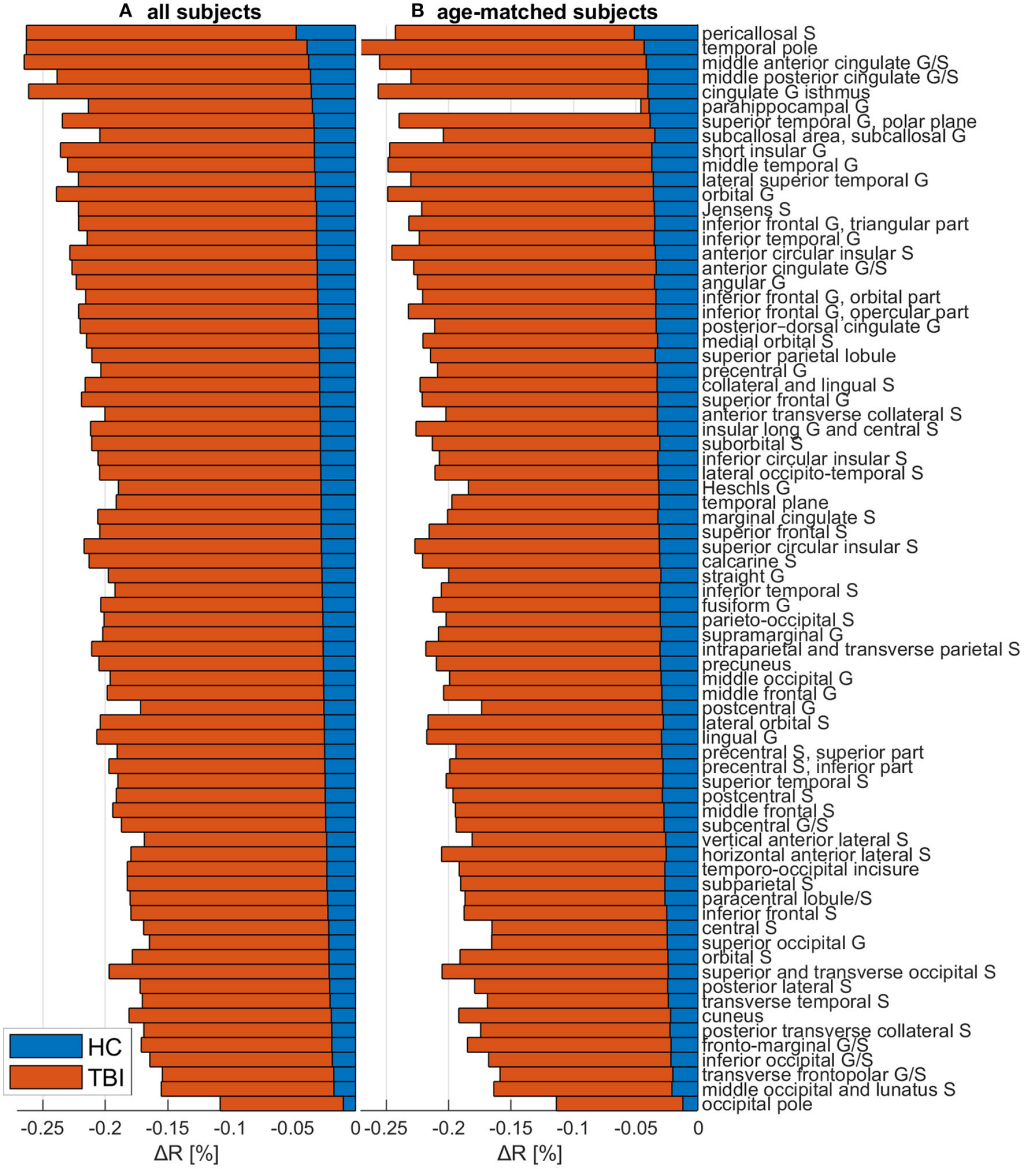
The change Δ*R* in the myelination ratio *R* for HCs (blue) and mTBI participants (orange), across both **(A)** the full cohort and **(B)** a sub-cohort containing only mTBI and HC subjects whose age ranges overlap. Δ*R* was calculated as Δ*R* = (*R*_*f*_−*R*_*b*_)/*R*_*b*_, where *R*_*b*_ is the *R* value at acute baseline and *R*_*f*_ is the *R* value at chronic follow-up. Δ*R* values were averaged across subjects and then across all cortical vertices within each region. Values are ordered in the ascending order of the full HC cohort's Δ*R* values. Abbreviations: G, gyrus; HC, healthy control; mTBI, mild traumatic brain injury; S, sulcus.

**Figure 7:**
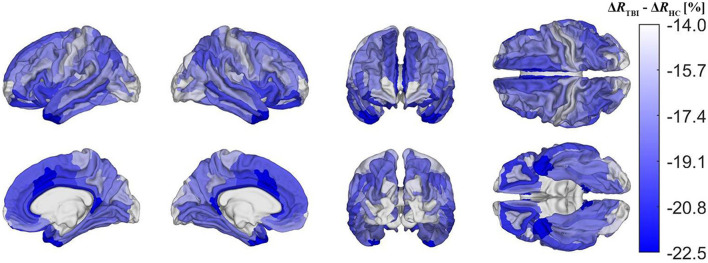
Cortical map of group differences in Δ*R*, corrected for multiple comparisons. These group differences convey deviations in mTBI-related demyelination from typical aging-related demyelination. Group differences were calculated as Δ*R*_*TBI*_−Δ*R*_*HC*_, where Δ*R*_*TBI*_ and Δ*R*_*HC*_ are Δ*R* for the mTBI and HC groups, respectively. Darker blue shades indicate that mTBI participants experience a greater percentage of myelin loss than HCs. The former experience a greater relative amount of demyelination in temporal, cingulate, and insular regions, but also a relatively smaller amount in the pre- and postcentral gyri, as well as in occipital regions.

Figure 5 depicts cortical maps of *u*(Δ*R*), according to the analysis described in the subsection on hypothesis testing. Whereas, both groups exhibit similar spatial patterns of cortical myelin loss, mTBI participants' demyelination is significantly more severe throughout the entire cortex (all *p* < 0.05). In HCs Figure 5A), regions with the smallest average decrease in *u*(*R*) include frontomarginal (1.9% loss on average), inferior occipital (1.86%), and transverse frontopolar (1.74%) gyri/sulci; middle occipital sulci (1.73%); and the occipital poles (0.98%). Regions with the largest average myelin loss include the temporal poles (3.88%); the middle-anterior (3.75%) and middle-posterior (3.60%) parts of the cingulate gyri and sulci; and the parahippocampal (3.45%), short insular (3.29%), and middle/superior temporal (3.28%) gyri. By contrast, in mTBI subjects (Figure 5B), myelin loss is more widespread and greater than in HCs. Across the follow-up period, all cortical regions are found to undergo decreases in *u*(*R*) larger than 10%. Structures with the greatest average myelin losses include the temporal poles (26.3%); the cingulate gyri/sulci (24.2%); and the orbital (23.9%), middle temporal (23.0%), angular (22.3%), inferior frontal (22.0%), superior frontal (21.9%), parahippocampal (21.4%), lingual (20.7%), and supramarginal (20.2%) gyri. These *u*(Δ*R*) values are listed in Figure 6A. Whereas all cortical regions undergo significantly more demyelination in mTBI subjects compared to HCs, the extent of this phenomenon varies across regions. The smallest difference between groups involves myelin at the occipital poles (9.90% more demyelination, on average, in mTBI participants than in HCs). Figure 7, obtained from the analysis described in subsection on visualization and comparison of results, highlights regions that undergo the greatest myelin loss in mTBI subjects compared to HCs: the middle-anterior (22.7%) and ventroposterior (22.6%) parts of the cingulate gyri and sulci; the temporal poles (22.4%); the pericallosal sulci (21.6%); and the orbital (20.7%), short insular (20.3%), and superior temporal gyri (20.1%).

### Regional Vulnerability to Demyelination and Atrophy

In mTBI participants, across the ~6-month follow-up period, occipital regions experience 22.0% less demyelination, on average, than the rest of the cortex, whereas cingulate, temporal, and insular regions experience more demyelination, on average, than the rest of the cortex (19.8, 16.5, and 14.3%, respectively). In HCs, across the follow-up period, occipital regions experience 32.5% less demyelination than the rest of the cortex, whereas cingulate, temporal, and insular regions experience more demyelination than the rest of the cortex (27.7, 27.4, and 18.4%, respectively). In mTBI subjects, across the follow-up period, no change in mean regional cortical thickness or volume survived multiple comparison correction (*q* < 0.05). HCs experienced no significant differences in mean regional cortical thickness or volume, except for a significant decrease in cortical thickness within the inferior part of the left precentral sulcus.

### Age Effects

Figure 6B, illustrating the outcome of the analysis described in subsection on age sensitivity analysis, highlights that regional *u*(Δ*R*) values computed across the full HC cohort are, overall, of similar magnitudes to those computed across the age-matched mTBI and HC subgroups. The ordering of regions is also similar, with the temporal and occipital poles consistently reported as being among the most and least demyelinated regions, respectively.

### Correlation of ***R*** With Cognitive Measures

At acute baseline, the average of *R* across the cortex was negatively correlated with WMS (*r* = −0.223, *p* = 0.047) and with IR (*r* = −0.248, *p* = 0.027); however, these findings did not survive correction for multiple comparisons. No other significant correlations were observed at either timepoint, nor were any correlations between cognition and *u*(Δ*R*_*TBI*_). The chronic follow-up *u*(*R*) across the anterior transverse temporal gyrus was correlated with the WMS score at acute baseline (*r* = −0.311, *p* = 0.005). There was also a significant correlation between the chronic follow-up *u*(*R*) across the anterior temporal gyrus and the IR score recorded at acute baseline (*r* = −0.311, *p* = 0.005). A similarly significant correlation was found between the chronic follow-up *u*(*R*) across the anterior parts of the cingulate gyrus and sulcus and the IR score recorded at acute baseline (*r* = −0.308, *p* = 0.006). These last two findings did survive correction for multiple comparisons. The correlations of *u*(*R*) with cognition are listed in Table 3.

**Table 3:** Spearman coefficients *r* and *p*-values for the correlation of relative myelin content *R* and cognitive scores for traumatic brain injury subjects, at 2 weeks and 6 months post injury.

	***R***								
	**ΔR**		**Acute baseline**		**Chronic follow-up**		**Significant regions**		
**BTACT**	***r***	***p***	***r***	***p***	***r***	***p***	**structure**	***r***	***p***
**(A) acute baseline**									
EVMI	0.005	0.960	−0.051	0.655	−0.142	0.211			
EVMD	−0.103	0.365	0.050	0.655	−0.135	0.233			
WMS	−0.129	0.253	−0.008	0.939	−0.223	**0.047**	TTG	-0.311	0.005^*^
IR	−0.048	0.674	−0.086	0.450	−0.248	**0.027**	ACC	-0.308	0.006^*^
							TTG	-0.311	0.005^*^
PS	0.071	0.533	−0.010	0.924	0.008	0.937			
VF	0.082	0.471	−0.025	0.823	0.006	0.956			
**(B) chronic follow-up**									
EVMI	−0.045	0.688	0.021	0.850	−0.162	0.152			
EVMD	0.001	0.989	0.002	0.981	−0.033	0.769			
WMS	−0.102	0.370	−0.042	0.707	−0.185	0.101			
IR	0.026	0.815	−0.001	0.997	−0.086	0.451			
PS	0.104	0.361	−0.040	0.724	0.022	0.841			
VF	0.170	0.133	−0.017	0.876	0.110	0.332			

*Bold indicates significant findings with p < 0.05, and*

* *indicates findings that survived the multiple comparisons correction. EVMI, episodic verbal memory immediate; EVMD, episodic verbal memory delayed; WMS, working memory span; VF, verbal fluency; IR, inductive reasoning; and PS, processing speed; ant. TTG, anterior transverse temporal gyrus; ACC, anterior part of the cingulate gyrus and sulcus*.

## Discussion

### Interpretation and Implications

According to this study, independently of age and sex, mTBI participants exhibit significantly lower average *R* values at 6 months follow-up than at the acute timepoint, and significantly more demyelination than HCs. This suggests not only that mTBI can have lasting effects on myelin levels, but also that the size of such effects increases with time within the first 6 months after injury. Reassuringly, our HC myelin maps largely resemble those of previous studies (12, 31). For example, unimodal sensory areas are highly myelinated whereas multimodal sensory and association areas are lightly myelinated. These trends are consistent with histological findings (31). In HCs, many regions with lower relative myelin content at baseline (e.g., temporal and frontal association areas) have been found to undergo relatively more demyelination than heavily myelinated regions (e.g., primary sensory areas), in agreement with findings from cross-sectional studies (20, 32).

According to the *z*-score formula Δ*z* = *z*_*TBI*_−*z*_*HC*_ (subsection on visualization and comparison of results), a lower *z*-score in mTBI participants (i.e., Δ*z* < 0) can reflect one of three scenarios: (A) *R*_*TBI*_ < *R*_*HC*_ and *u*(*R*_*TBI*_) = *u*(*R*_*HC*_); (B) *R*_*TBI*_ = *R*_*HC*_ and *u*(*R*_*TBI*_) > *u*(*R*_*HC*_); (C) both (A) and (B). The preponderance of the evidence suggests that myelin content decreases after mTBI (2, 6). Therefore, when Δ*z* < 0, *u*(*R*_*HC*_) > *u*(*R*_*TBI*_). At baseline, lateral occipital regions exhibit lower *z*-scores in mTBI participants than in HCs (i.e., Δ*z* < 0, Figure 4) such that, at baseline, lateral occipital regions likely have lower *R* in mTBI subjects than in HCs. Conversely, in both HCs and mTBI participants, these occipital areas were also found to be among the *least* vulnerable to demyelination based on the percentage difference in myelin *between timepoints* in each region (Figure 5). At baseline, temporal, cingulate, and insular regions have higher *z*-scores in mTBI subjects than in HCs, indicating that these areas may be among the *least* vulnerable to acute demyelination. However, these regions are also among the *most* vulnerable to chronic demyelination. Thus, our results indicate that cortical regions can differ significantly in their vulnerability to acute vs. chronic mTBI. The differential vulnerability of brain regions to demyelination in acute vs. chronic mTBI suggests that the mechanisms underlying *acute* post-traumatic demyelination differ, at least in part, from those resulting in *chronic* demyelination.

Although demyelination is significantly more severe in chronic mTBI than in typical aging, the spatial pattern of regions most or least vulnerable to demyelination overlaps substantially across these groups. Such similarities may indicate that, typically, these brain regions are similarly vulnerable to both aging- and mTBI-related demyelination mechanisms, or that demyelination is caused by similar factors in both groups. This supports the hypothesis that chronic mTBI-related demyelination is linked to secondary mechanisms of brain damage, which typically include neuroinflammation and excitotoxicity that are also manifest in typical aging (33–35). Furthermore, the regions most vulnerable to demyelination across both groups (e.g., insular, temporal, and cingulate cortices) overlap greatly with those that undergo significantly more demyelination after mTBI than is typical (Figure 7). Conversely, the regions least vulnerable to demyelination across both groups (e.g., occipital, frontomarginal) undergo significantly less demyelination than the average amount expected after mTBI. This suggests the testable hypothesis that chronic mTBI effects on myelin may compound non-linearly in these areas, possibly due to interplay between various secondary injury mechanisms.

The paucity of significant correlations between cognitive scores and *R* values is likely influenced by cognitive recovery (36, 37). Post-traumatic recovery of myelin occurs to some degree in WM (9) but is understudied in the GM. Primary injury mechanisms can result in axonal damage and cell death before remyelination can begin (38), and studies of intracortical remyelination in multiple sclerosis reveal that, even when myelin recovery occurs, the patterns of myelination may be altered pathologically (39). The negative associations between IR, WMS, and *R* are surprising, but likely reflect that IR and WMS are not exclusively dependent on focal myelin profiles. IR and WMS are cortically distributed processes that require communication across multiple GM areas via WM connections. Thus, it is possible that *R* reflects short-range local communication better than long-distance cortico-cortical processing of stimuli or neurally encoded information.

Occasionally, the spatial pattern of mTBI-related demyelination mapped here deviates from that observed in typical aging-related demyelination, e.g., in the case of sensorimotor cortex. Pre- and postcentral gyri (which harbor the primary motor and somatosensory cortices, respectively) experience substantially more *typical aging-related* demyelination, but substantially less *mTBI-related* demyelination than most other regions. In this respect, there are notable similarities between mTBI- and AD-related demyelination patterns. Although the latter remains understudied in the cortex, it is known that white matter tracts linking temporal areas to the rest of the cortex undergo significantly more demyelination in AD, whereas tracts proximal to motor and somatosensory cortex are relatively spared (40). Furthermore, AD causes excessive cortical thinning of orbitofrontal, anterior temporal, parietal, cingulate, and insular regions (41–43), while sparing somatosensory regions (44). This cortical thinning pattern mirrors the demyelination pattern documented here in chronic mTBI (Figure 5B). AD-related cortical thinning is partly due to neuroinflammation of the cortex (45), which is known to be both a cause and a consequence of demyelination, across both AD and TBI (33, 46). In this context, our findings suggest that there is substantial overlap between the brain's spatial patterns of vulnerability to (A) AD-related cortical thinning, (B) AD-related white matter demyelination, and (C) mTBI-related intracortical demyelination. Thus, because mTBI increases the risk of AD and related dementias (47, 48) through uncertain mechanisms, further study on how post-traumatic demyelination patterns can translate into higher AD risk is warranted.

It is possible that edema lowers *R* after mTBI. Edema may cause hyperintensity on *T*_2_-weighted MRI, leading to lower *R* values, and both vasogenic as well as cytotoxic edema can become manifest after mTBI (49). Cortical thickening can be a correlate of post-traumatic edema, because this latter phenomenon is often due to acute inflammation leading to intra- and extracellular fluid accumulation that translates into larger regional volumes and thicker cortex despite frequent loss of neurons (50). Given that the mTBI subjects in this study experienced no significant change in cortical thickness or volume across the follow-up period, edema may not have played a substantial role in causing reductions in *R* across ISIs.

It has been argued that *R* may correlate with neuroanatomical features other than myelin. Correlations between *R* and dendrite density, axon caliber, and cell protein size have been reported, as has a negative relationship between *R* and mitochondrion gene variants (51, 52). The latter association may imply abnormally high mitochondrial energetics in brain regions with low *R* in this study (e.g., parts of the temporal, cingulate, and insular cortex). Abnormally high mitochondrial energetics are known to contribute to neurotoxicity (53). On the other hand, the association between protein size and *R*, which is partially related to the negative correlation between *R* and proteasome-expressed genes (52), could indicate malignant proteolysis, which has been described as a primary effect of TBI that accompanies traumatic axonal injury (54). Thus, *R* may correlate with markers of both primary and secondary injury after mTBI. Future studies should explore complementary measurements that can refine the interpretation of *R* values and their changes across the brain.

Whether based on the full cohort or on the age-overlap subgroups, our within-group Δ*R* comparison is qualitatively and quantitatively consistent. This suggests that our regression model was effective in partialing out the statistical effect of age, despite our use of a relatively younger mTBI cohort and of a relatively older HC cohort. This sensitivity analysis also suggests that age distribution discrepancies between HCs and mTBI participants did not have a substantial effect on our ulterior analysis.

### Comparison With Other Studies

The relative amount of post-traumatic demyelination reported in this study is noteworthy. Although diffusion MRI studies of mTBI do not typically report brain circuitry changes of similar magnitudes (55, 56), such studies focus on WM changes rather than on GM changes due to the far more isotropic diffusion of water within GM compared to WM (19). Furthermore, the extent to which diffusion MRI measures of WM integrity are related to myelin content in the GM is poorly understood, thus precluding interpretation of our findings relative to diffusion MRI studies. Magnetization transfer ratio studies similarly tend to find smaller effects than ours, but these studies are also focused primarily on WM (57). One of the few imaging other measures to survey intracortical myelin is the macromolecular protein fraction (MPF). To our knowledge, only one study has used MPF to investigate myelin content after human mTBI. That study reported MPF reductions of 18–36% in precentral, anterior cingulate, medial orbital, subcallosal, lingual, superior frontal, and inferior frontal gyri (58). However, these reductions were observed across periods much longer than the typical ISI in our study (i.e., ~10 years versus ~6 months), and in persons who had sustained several mTBIs rather than just one, as in our case. Thus, interpretation of our results relative to available MPF findings requires further studies.

It is illustrative to compare the findings in our HC cohort to those in a cross-sectional lifespan study of *R* by Grydeland et al., who studied an HC cohort different from ours (20). Despite similar findings in terms of regional patterns of demyelination (e.g., greater demyelination in cingulate/temporal cortex, less demyelination in sensory cortex), Grydeland et al. reported more gradual declines in *R* than are seen in our study at certain cortical regions. Discrepancies between the two studies may be due to methodological differences, such as our inclusion of bias field correction and our mapping of demyelination in a longitudinal cohort rather than cross-sectionally.

Importantly, we must remind the reader that the parameters of the mathematical function describing the relationship between *R* and true cortical myelin content are not known with high precision. For this reason, we cannot determine whether our computed values of Δ*R* reflect the true percentage change in cortical myelin that would be determined by direct measurement. It is possible, for example, that Δ*R* scales linearly with true myelin content, or that this scaling is non-linear. However, because the precise coefficients of either model are unknown to us, our reported Δ*R* values may convey a measure of relative demyelination extent rather than true values of myelin content change.

Only one other study known to us (15) calculated *R* in persons with mTBI, finding a significant negative association between *R* and the *number* of mTBIs sustained by participants. The negative association was strongest in lateral occipital areas, which is inconsistent with our findings on regional vulnerability to *chronic* mTBI (Figure 5B), but consistent with those on *acute* mTBI (Figure 4). This discrepancy may imply a shared mechanism of demyelination between repeat and acute mTBI, but further research is needed to clarify the matter. One interpretation is that primary injury mechanisms drive acute post-traumatic demyelination and are upregulated in repeat mTBI because of multiple distinct injuries (55). Future studies should integrate *T*_1_- and *T*_2_-weighted MRI with other approaches that can distinguish primary from secondary injury effects upon the cortex.

### Limitations

HCs and mTBI participants were not imaged as part of the same study. Thus, on average, HCs are typically older than mTBI participants and their ISIs are longer as well. Although these group differences were accounted for in the statistical analysis, we acknowledge that it would have been preferable for the HC and mTBI groups to be better matched according to these variables. In our HCs, 30% had at least one ε4 allele, compared to ~13% in the general population (59). The ε4 genotype is known to be associated with higher AD risk and more severe demyelination (60). Thus, differences in genotype between cohorts could partly explain some group differences reported here, particularly if the mTBI group had fewer ε4 allele carriers than the HC group. Because the APOE genotype of mTBI participants was unknown to us, its statistical effect could not be accounted for. We acknowledge this as a limitation of our analysis; future studies should quantify whether and how APOE genotype affects post-traumatic demyelination.

The drawbacks of estimating myelin content from the ratio *R* of *T*_1_/*T*_2_-weighted MRIs should be acknowledged. Although *R* is significantly correlated with myelin-related gene expression in the cortex, *R* is correlated more strongly with axon caliber and oligodendrocyte markers (52) and the myelin water fraction index has been proposed by Arshad et al. (61) as a preferable index of myelin content, although these authors compared *R* and myelin water fraction only from the standpoint of their abilities to quantify myelin in the white (rather than gray) matter. A later study (62) found that myelin water fraction correlates poorly with *R* in white matter, but more strongly in gray matter, such that our use of *R* in this study of *cortical* myelin is likely justified. Aside from these considerations, however, we acknowledge that estimating myelin content from the ratio of *T*_1_/*T*_2_-weighted MRIs can be affected by intensity scaling disparities across individuals, scanners (61), and possibly even across neurological conditions (63), although evidence for this latter effect is equivocal (64). Specifically, Pelkmans et al. found higher *R* values in AD compared to cognitively normal persons. However, as they discuss, these results were likely influenced by the iron deposition that accompanies AD. Iron is reflected as hyperintensity in *T*_1_images, resulting in an increased *R* value. mTBI, on the other hand, likely does not cause the same level of cortical iron deposition within 6 months after injury. Acute iron deposition in mTBI is mostly heme-bound, manifesting as cerebral microbleeds (65), which are typically small and few (66), although their incidence increases with age (67). Non-heme iron accumulation after mTBI is understudied in humans but has, thus far, been found only near ventricles, not in cortical GM (65, 68).

Because follow-up scans were registered to baseline *T*_1_-weighted scans for each subject, the follow-up *T*_1_-weighted scans were subjected to more interpolation than baseline *T*_1_-weighted scans. Due to the high sampling percentage used by the registration procedure and to the high fidelity of the registration between baseline and follow-up scans, this discrepancy is likely to have had very limited effect upon MRI intensities. However, we acknowledge that applying an identical interpolation to baseline *T*_1_-weighted scans may have been preferable. Although mTBI and HC participants were not scanned as part of the same study, the scanner parameters were similar across mTBI and HC groups. Our previous studies (66, 69) on a sample that overlapped substantially with that in the current one quantified differences in aging between mTBI and HC groups and found no significant differences between these groups pertaining to the statistical effects of sequence parameters and scanner types. Additionally, comparisons of Δ*R* were unlikely to be impacted by this confound, as scanner parameters were identical across follow-up for all subjects. Nevertheless, we acknowledge that scanning both mTBI and HC participants using the same scanner and sequence parameters would have been preferable. The *z*-score analysis implemented for the baseline comparison circumvented the issue by comparing the *distribution* of *R* values between groups, but we acknowledge that this analysis relied on the assumption that the effects of scanner parameters on *R* were mainly consistent across the cortex. If scanner parameters had significant variation in their effects on *R* at different cortical regions, that could potentially complicate the interpretation of acute mTBI effects on relative myelin content. This confound is expected to have had minimal impact, especially due to the nature of the *T*_1_*/T*_2_-weighted ratio, which increases contrast for myelin specifically, thereby reducing the variation in response to other tissue features that *T*_1_- or *T*_2_-weighted imaging may exhibit alone (12, 14). The regression model partialed out the statistical effect of within-group variation related to scanner differences, a confound that only impacted HCs. The cortical patterns of *R* was very similar across HCs scanned using GE and Siemens scanners, and there was no significant difference (*p* < 0.05) in either μ(*R*) or σ(*R*) across scanner manufacturers. The sequence parameters were consistent across scanner manufacturers.

## Conclusion

Mapping the spatiotemporal profile of demyelination after mTBI is clinically relevant. This study suggests that chronic mTBI-related intracortical demyelination has a distinct spatial profile from that observed in the acute phase of mTBI. Furthermore, post-traumatic demyelination is substantially more severe than typical aging-related demyelination. These and related findings of our study have implications for elucidating the mechanisms whereby mTBI-related demyelination occurs. Because post-traumatic cortical demyelination can affect clinical outcomes and may even reflect the risk for neurodegenerative diseases like AD, future studies should further investigate this phenomenon relative to other factors like age, sex, APOE genotype, cognitive function, and other biological factors. Future research should also investigate the association between our MRI measures and independent biomarkers of demyelination, e.g., serum levels of neurofilament light chain protein.

## Data Availability Statement

Primary data generated and/or analyzed during the current study are available subject to a data transfer agreement. At the request of some participants, their written permission is additionally required in some cases.

## Ethics Statement

The studies involving human participants were reviewed and approved by Institutional Review Board at the University of Southern California. The patients/participants provided their written informed consent to participate in this study.

## Author Contributions

SM: study concept and design, algorithm implementation, data acquisition and analysis, and contributions to manuscript. NC: study design, data analysis and interpretation, and contributions to manuscript. VN: algorithm implementation and data acquisition and analysis. PI: data analysis and contributions to manuscript. AI: study design, manuscript revision and review, data analysis and interpretation, and implementation of statistical analysis. All authors contributed to the article and approved the submitted version.

## Funding

This study was supported by the National Institutes of Health Grant R01 NS 100973 to AI, by the US Department of Defense contract W81XWH-18-1-0413 to AI, by a Hanson-Thorell Family Research Scholarship, and by the James J. and Sue Femino Foundation. Data collection and sharing for this project was funded by the Alzheimer's Disease Neuroimaging Initiative (ADNI) (National Institutes of Health Grant U01 AG024904) and DOD ADNI (Department of Defense Award Number W81XWH-12-2-0012). ADNI is funded by the National Institute on Aging, the National Institute of Biomedical Imaging and Bioengineering, and through generous contributions from the following: AbbVie, Alzheimer's Association; Alzheimer's Drug Discovery Foundation; Araclon Biotech; BioClinica, Inc.; Biogen; Bristol-Myers Squibb Company; CereSpir, Inc.; Cogstate; Eisai Inc.; Elan Pharmaceuticals, Inc.; Eli Lilly and Company; EuroImmun; F. Hoffmann-La Roche Ltd and its affiliated company Genentech, Inc.; Fujirebio; GE Healthcare; IXICO Ltd.; Janssen Alzheimer Immunotherapy Research & Development, LLC.; Johnson & Johnson Pharmaceutical Research & Development LLC.; Lumosity; Lundbeck; Merck & Co., Inc.; Meso Scale Diagnostics, LLC.; NeuroRx Research; Neurotrack Technologies; Novartis Pharmaceuticals Corporation; Pfizer Inc.; Piramal Imaging; Servier; Takeda Pharmaceutical Company; and Transition Therapeutics. The Canadian Institutes of Health Research is providing funds to support ADNI clinical sites in Canada. Private sector contributions are facilitated by the Foundation for the National Institutes of Health (www.fnih.org). The grantee organization is the Northern California Institute for Research and Education, and the study is coordinated by the Alzheimer's Therapeutic Research Institute at the University of Southern California. ADNI data are disseminated by the Laboratory for Neuro Imaging at the University of Southern California. The funders were not involved in the study design, collection, analysis, interpretation of data, the writing of this article or in the decision to submit it for publication.

## Conflict of Interest

The authors declare that the research was conducted in the absence of any commercial or financial relationships that could be construed as a potential conflict of interest.

## Publisher's Note

All claims expressed in this article are solely those of the authors and do not necessarily represent those of their affiliated organizations, or those of the publisher, the editors and the reviewers. Any product that may be evaluated in this article, or claim that may be made by its manufacturer, is not guaranteed or endorsed by the publisher.

## Abbreviations

ADNI: Alzheimer's Disease Neuroimaging Initiative
APOE: apolipoprotein E
BTACT: brief test of adult cognition by telephone
df: degrees of freedom
DWI: diffusion weighted imaging
EVMD: episodic verbal memory delayed
EVMI: episodic verbal memory immediate
FS: FreeSurfer
GM: gray matter
ISI: interscan interval
IR: inductive reasoning
MPF: macromolecular protein fraction
MCI: mild cognitive impairment
mTBI: mild traumatic brain injury
PET: positron emission tomography
PS: processing speed
*T*_*E*_: echo time
*T*_*I*_: inversion time
*T*_*R*_: repetition time
VF: verbal fluency
WM: white matter
WMS: working memory span.
